# A review of the neuroscience of religion: an overview of the field, its limitations, and future interventions

**DOI:** 10.3389/fnins.2025.1587794

**Published:** 2025-08-19

**Authors:** Harrison M. Carvour, Anna K. Radke, Nathan S. French

**Affiliations:** ^1^Department of Psychology, Miami University, Oxford, OH, United States; ^2^Department of Comparative Religion, Miami University, Oxford, OH, United States

**Keywords:** neuroscience of religion, brain imaging, traumatic brain injury, pharmacology, religious phenomena, religious theory

## Abstract

The neuroscience of religion (NoR), a field that studies various neural mechanisms and functions alongside their correlational relationships with religious phenomena, holds immense potential for development. Standard research methods, including brain imaging techniques and lesion studies, establish relationships between brain functions and religious practices, beliefs, and experiences. Despite increasing interest in this field since the start of the century, NoR has many limitations regarding the technology presently used, particularly in investigating specific aspects of religious behaviors regarding ritualized movements. However, using technologies not presently used in the field, such as functional near-infrared spectroscopy (fNIRS), along with applying other religious theories, could allow researchers to examine other behaviors, expand their understanding of religion, and bypass current limitations. Therefore, this review paper critically examines NoR, including discussions on methodologies and conclusions from the field, the application of theories of religion currently used, limitations of research methods, the potential incorporation of other theories of religion in the future, and the utilization of other technologies not presently employed in the field, possibly opening new avenues for inquiry and analysis.

## 1 Introduction

Neuroscience of religion (NoR), also known as neurotheology or spiritual neuroscience, is a field of neuroscientific research investigating the correlational relationships between brain functions and religious phenomena (Sayadmansour, 2014). Investigations seek to find neural correlates in structures, circuits, and signaling pathways relating to religious practice, rituals, beliefs, and experiences (Yaden et al., 2016). Research in this field could also prove vital in understanding the neural mechanisms behind disorders and compulsive behaviors in the context of religion, such as scrupulosity, which is commonly referred to as religious obsessive-compulsive disorder and is characterized by obsessions and compulsions around moral or religious issues and behaviors (Miller and Hedges, 2008). Despite being a relatively new line of research in neuroscience, entering its third decade of work, discoveries in the field have secured ongoing interest (Boyer, 2008; Sayadmansour, 2014; Yaden et al., 2016). The development of brain imaging technologies has opened new avenues of inquiry for researchers (Jastrzębski, 2018). These include brain imaging studies examining a variety of religious expressions, including Franciscan nuns praying, Buddhists meditating, and Christian-identifying military veterans who, while recovering from traumatic brain injury (TBI), have presented new possibilities for theorizing the neural correlates of religious behaviors (Sim and Tsoi, 1992; Boyer, 2003; Brown, 2017; Zhong et al., 2017). Research observing these populations suggests several prefrontal cortical regions may be critical regulators of empathetic responses to perceived personal relationships with a higher power (Zhong et al., 2017; Grafman et al., 2020). Other research that recorded interactions between the prefrontal cortex (PFC) and the nucleus accumbens (NAc) in devout Mormons suggests the importance of the reward pathway in attentional and emotional behaviors in religious contexts (Ferguson et al., 2018). So, despite being a younger neuroscience field, the research has yielded significant evidence for further exploration.

However, as with any scientific field, there are outer boundaries to the field of NoR. As the field developed, critical interventions exposed specific assumptions and identified challenges to its methods, leading to concerns regarding the validity of its research goals and findings (Cunningham, 2011; Vliegenthart, 2011). Behavioral neuroscientists argue that religious behaviors cannot be recorded empirically to establish neural correlates (Newberg, 2010; Cunningham, 2011). Academic researchers of religion argue that the research methodologies used in the field do not accurately represent religious behavior or phenomena. In particular, academic researchers of religion critique NoR for its lack of incorporation of theories into its research to help explain or justify the phenomena observed. Academic researchers of religion also critique NoR for its definitions of religion, religious experience, or religious phenomena (Cunningham, 2011; Vliegenthart, 2011; Jastrzębski, 2018).

Due to the concerns of behavioral neuroscientists and academic researchers of religion, this article will summarize the current state of research into NoR and suggest future directions. Topics in this article will include descriptions of standard research methods used in the field, such as brain imaging/recording techniques that include electroencephalography (EEG), functional magnetic resonance imaging (fMRI), and positron emission tomography (PET), along with TBI, psychedelic, and pharmacological research. Such methodological descriptions will also include details on the kinds of religious behaviors these methods aim to examine. Next, descriptions of findings and results relating to these different methodologies will be presented. Following these findings, this article will discuss the perceived limitations to the field of NoR, primarily revolving around how the current technologies used limit the kinds of behaviors researchers investigate, particularly those revolving around movement and ritualization. Finally, this article will discuss possible future directions to counter these limitations in this field. These future directions include the application of additional theories to conceptualize religion, such as those from Max Weber and Henri Lefebvre, and the use of brain imaging and recording technologies that permit investigation of physical activity and flow states to further explore the neural bases of religion and ritualized embodiment.

## 2 Methods in the field

### 2.1 Religious behaviors

A complete understanding of the techniques used to study NoR requires an introduction to the religious behaviors investigated. Exemplars of studies of such behaviors examine belief or ritual, making assumptions about the definition and essential nature of both (Yaden et al., 2016). In these studies, beliefs are commonly defined as a state of mind in which a person feels oneness with the world, a deity, or supernatural forces, addressing the existence of a deity or supernatural forces and understanding the purpose of one’s existence. Rituals are defined as involving intentional engagement with an activity outside of the structure of daily life (Yaden et al., 2016). Rituals and beliefs must work together to create an emotional state that allows for investing in symbols of sacred importance (Durkheim, 2001). Therefore, observed religious behaviors for this research typically involve prayer or meditation, listening to or reading a sacred text, viewing religious objects, describing one’s religious beliefs, or taking a religiosity survey or questionnaire on a Likert Scale (Azari et al., 2001; Yaden et al., 2016).

When studying religion, whether in neuroscience or any other field, it is essential to establish how religion is being defined. For starters, it is vital to consider that religion can be interpreted as the creation of a scholar’s study out of means to categorize and study belief and ritual systems across regions, communities, and institutions; essentially that the concept of religion only exists insomuch as how it is defined (Smith, 1982). Many definitions exist within the scope of comparative religious study. There is no formalized definition of religion that academic researchers of religion agree upon, though some theories hold more influence than others (Zinnbauer et al., 1997; Larson et al., 1998; McKinnon, 2002). However, in scientific fields, it is typical to establish a single, formalized definition that all researchers use to establish a unified understanding.

Regarding NoR, the definition utilized by the field was formalized by Andrew Newberg, whose theories are often cited (Jastrzębski, 2018; Rosmarin et al., 2022). As part of his attempt to define religion, Newberg relies on theories from William James and Emile Durkheim. Citing James, Newberg describes religion as believing in a god and trying to live by divine commandments (James, 1988). With Durkheim, Newberg affirms a social aspect of religion as a moral community featuring shared beliefs at its core (Durkheim, 2001). Durkheim also conceptualized religiously defined behaviors as two distinct categories: belief and ritual (Durkheim, 2001; Yaden et al., 2016). Therefore, the conceptualizations that NoR researchers use to describe religious behaviors use this Durkheimian model. Newberg also includes more recent theories from Michael Argyle and Benjamin Beit-Hallahmi, who describe religion as beliefs about a supernatural entity and shared practices and rituals (Argyle and Beit-Hallahmi, 2013). By utilizing these sources, Newberg broadly defines religion as a system of beliefs about supernatural phenomena and practices intended to acknowledge or interact with those phenomena. The most common conceptions of religion reflect the worship of divine beings as literal entities or representations of abstract forces. Social organizations, including facilities of worship and official councils of religious authority, often maintain the beliefs and practices utilized in religions (Yaden et al., 2016). However, as stated previously, there are many definitions of religion amongst academic researchers, and these theories have diverse ideas that are not expressed in how neuroscientists conceptualize religion. For instance, other scholars define religion relative to geography, movement, and color (Long, 1999; Tweed, 2009; Taussig, 2010).

Another approach regarding research into NoR that is frequently utilized in the field is the Theory of Mind (ToM) (Grafman et al., 2020). The concept behind ToM is that people can attribute mental states to others who are different from themselves. Utilizing ToM, researchers typically attribute it or pair it with empathy. Researchers established ToM and empathy as bases to theorize an evolutionary origin of religions as a mechanism for survival and promoting prosocial behavior (Tang et al., 2015; Norenzayan et al., 2016; Chung, 2018). In such discussions, researchers describe religious beliefs and behaviors as complex socio-cognitive phenomena that require higher social functioning abilities and, therefore, link brain regions associated with such functions to the evolutionary origin of religions (Kapogiannis et al., 2009; Grafman et al., 2020). Researchers also discuss ToM as necessary for participants to interpret the actions of what is perceived as a supernatural force, entity, or deity. Most of the research that discusses ToM comes from studies examining TBI patients who have received cortical damage to regions associated with higher cognitive functions relating to social and empathetic behaviors (Kapogiannis et al., 2009; Modestino et al., 2016; Zhong et al., 2017; Grafman et al., 2020; Cristofori et al., 2021; Cristofori et al., 2022).

### 2.2 Brain imaging and recording

Electroencephalography (EEG) is one technique through which researchers investigate the neural correlates of religious behaviors. EEG measures electrical activity in the brain using electrodes attached to the scalp, which are recorded as waves. These waves are then analyzed to measure their amplitude and frequency to determine brain activity or states of consciousness (Banquet, 1973; Hebert and Lehmann, 1977). With recent developments in this technology, researchers are better equipped to identify the source of a waveform of interest through source localization. EEG source localization is a more complex process that involves estimating the distribution of neuronal sources that generate activity on the scalp (Muñoz-Gutiérrez et al., 2018). With such an advancement, EEG activity can be localized to certain anatomical regions, though these are broad and primarily cortical (Muñoz-Gutiérrez et al., 2018). The use of EEG in investigating neural correlates of religious behaviors began with early studies measuring the electrical activity in the brain during meditation practices (Banquet, 1973; Hebert and Lehmann, 1977). EEG has proven to be a valuable research tool for investigations into the neural correlates of religious behavior, especially in meditative practices, due to this technique’s relatively non-invasive recording methods (Lehmann et al., 2001; Aftanas and Golocheikine, 2002; Travis and Arenander, 2004; Newberg, 2020). This allows EEG to be especially useful in recording meditative and ritualistic states, enabling participants to enter such mindsets while limiting the amount of external stimuli that could impede reaching such states. EEG also offers opportunities for investigations into ritual movement, based on this technology utilizing a headset of electrodes connecting wires to a system and program that is not as restrictive as other brain imaging and recording techniques (Banquet, 1973; Hebert and Lehmann, 1977). However, despite this technology offering a chance to record brain activity from such behaviors, research has yet to explore ritual movement using EEG (Newberg, 2020).

Functional magnetic resonance imaging (fMRI) is another approach to investigating the neural correlates of religious behaviors. Using fMRI allows researchers to evaluate neural activity associated with various states, such as sensory stimulation, motor function and coordination, language, and higher cognitive functions (Newberg, 2020). fMRI measures changes in cerebral blood flow during specific tasks, providing researchers with a proxy for neural activity across multiple brain regions (Newberg, 2020). fMRI also has excellent temporal resolution, allowing researchers to obtain multiple images over short periods. Therefore, researchers can examine the performance of multiple behaviors during the scanning session, such as a sequence of prayers (Newberg, 2020).

Positron emission tomography (PET) is another form of brain imaging that provides information about brain function during a task or other behavior of interest (Newberg, 2020). The significant difference between PET imaging and fMRI is that PET imaging requires the injection of a radioactive tracer that, depending on the type used, can help measure a variety of functional parameters, such as cerebral blood flow, cerebral glucose metabolism, and receptor occupancy in neurotransmitter systems (Newberg, 2020). This latter capability means PET can offer unique insights into the neural correlates of religious behaviors by examining the involvement of neurotransmitters along with brain regions.

### 2.3 Brain lesions and injury

An intriguing direction in NoR is examining the effects of traumatic brain injury (TBI) or other brain disorders on religious behaviors (Ferguson et al., 2022). Studies that have examined religious behaviors in these instances have investigated participants with epilepsy associated with the temporal lobe, hippocampus, and amygdala, along with parietal and frontal lobe damage (Geschwind, 1983; Ogata and Miyakawa, 1998; Wuerfel et al., 2004; Devinsky and Lai, 2008; Urgesi et al., 2010; Asp et al., 2012; Zhong et al., 2017). Most of the findings in TBI and religion studies have relied on a specific subject population. The participants in these studies have typically been Vietnam War veterans who had suffered brain lesions related to their experiences in warfare. While Vietnam War veterans are a unique population that have been investigated in this research, studies have yet to be conducted on other veteran populations that have served in different conflicts. For instance, how might veterans who have suffered a TBI while on duty in the Gulf War or the War on Terror impacted their religious identity? As stated previously, ToM is a common attribute discussed in this research (Zhong et al., 2017; Cristofori et al., 2021; Cristofori et al., 2022). ToM is mainly recruited in TBI-related research due to where brain lesions have occurred. TBI research investigating the neural correlates of religious behavior primarily uses participants who have experienced brain lesions in the temporoparietal junction (TPJ), the parietal lobe, and the prefrontal cortex (PFC). These regions are essential for higher cognitive functions, such as empathetic behaviors (Cristofori et al., 2021; Cristofori et al., 2022). Therefore, these studies examine how brain lesions alter empathetic or ToM-related behaviors to investigate how people understand religious deities and interact with those inside and outside their religious communities (Lawrence, 1997; Cristofori et al., 2021; Cristofori et al., 2022).

### 2.4 Pharmacology

Another intriguing direction in NoR is incorporating pharmacological methods into the research. One such pharmacological method includes the use of psychedelics. It has long been observed that psychedelic drugs have been used in ritualized settings to elicit experiences described as religious or spiritual, such as ayahuasca and peyote (Newberg, 2020). By understanding the neuropsychopharmacology of such compounds with religious or spiritual experiences, researchers can establish correlates relating to brain regions, circuits, and neurotransmitter systems that can elucidate religious or spiritual experiments. While several studies have aimed to induce religious or spiritual experiences through the administration of psychedelic compounds, research on this topic needs to be explored further (Vollenweider et al., 1997; Vollenweider et al., 1999; Vollenweider et al., 2000; Griffiths et al., 2006; Newberg, 2020).

Neuropharmacological correlates of religious behavior have also been studied by administering receptor agonists or antagonists. Utilizing such a technique allows researchers to discern how specific neurotransmitter systems can impact certain behaviors using centrally acting drugs, typically combined with brain imaging or recording techniques (Sim and Tsoi, 1992). Analysis of neurotransmitter and neurohormone levels in blood plasma, blood serum, and urine samples following engagement with religious behaviors has also yielded insights (Newberg, 2020). After participating in religious behaviors, more direct assessments of brain neurotransmitter levels have been achieved through microdialysis and analysis of participants’ cerebrospinal fluid (CSF) (Newberg, 2020).

## 3 Discoveries

### 3.1 Cognition and behavior

#### 3.1.1 Theory of mind

Using a combination of brain imaging, false-belief tasks, and religious belief statements involving God’s perceived level of involvement, emotion, and intentions, researchers have implicated the medial prefrontal cortex (mPFC), superior temporal sulcus, TPJ, precuneus, and inferior frontal gyrus (IFG) in ToM-related behaviors and cognitions (Grafman et al., 2020). In one study, statements involving the perception of God’s lack of involvement activated these brain networks involved with understanding an agent’s actions, processing emotions, and self-relevance, which are essential for comprehending the intentions of another agent (Kapogiannis et al., 2009). Another study discovered that these regions play a crucial role in religious beliefs by influencing the activity of downstream non-ToM brain regions (Kapogiannis et al., 2014). A pathway from the right IFG, a crucial brain region for ToM, due to its role in understanding others’ mental states, modulates the dorsomedial prefrontal cortex (dmPFC) and precuneus while religious subjects verified statements concerning the perceived involvement of God in their daily lives (Kapogiannis et al., 2014).

Other studies with highly religious-identifying individuals have revealed that these regions are also active during prayer (Schjoedt et al., 2009). This finding demonstrates that personal prayer activates a network of brain regions involved with social cognition, including the temporopolar region, TPJ, precuneus, and MPFC. This activation emphasizes the similarity between praying to God and non-religious human communication, as prayer involves viewing God as an intentional agent of reciprocation (Schjoedt et al., 2009).

#### 3.1.2 Semantic processing and storage

An fMRI study examining sacred values tied to religious or moral principles and their impact on behavior found that these values influence behavior by activating and recalling semantic rules rather than practically assessing costs and benefits (Berns et al., 2012). In this study, participants were asked whether they would compromise their beliefs for financial gain, revealing the sacred values they were unwilling to relinquish. The processing of these sacred values activated the left TPJ and left ventrolateral prefrontal cortex (vlPFC), both of which are known for their involvement in retrieving and processing semantic rules (Berns et al., 2012). Other studies have demonstrated that recalling religious beliefs stored in semantic and episodic memory can trigger emotional reinforcement of belief or disbelief. This was observed through the activation of the anterior insula, amygdala, and other associated structures involved in processing emotionally significant stimuli (Harris et al., 2008; Harris et al., 2009). Of course, other brain regions, such as the IFG, dmPFC, and precuneus, are involved with processing and episodic memory (Grafman et al., 2020).

#### 3.1.3 Multisensory integration

People may experience moments of “spiritual enlightenment,” apart from following and contemplating religious teachings. These experiences, often seen as sacred, have similarities with artificially induced transcendence that involves the integration of far-reaching multisensory processes (Lifshitz et al., 2019). Psychoactive drugs or certain environmental stimuli can bring about this state, with the functioning of the parietal lobe and the involvement of the frontoparietal attention network (Lifshitz et al., 2019).

#### 3.1.4 Cognitive control

Research on the connection between religious beliefs and cognitive control has its roots in the observation of religious episodes in patients with right temporal lobe epilepsy (Devinsky and Lai, 2008). Additionally, the analysis of associations among religious beliefs, cognitive processing, and brain damage in male Vietnam combat veterans with penetrating TBIs has furthered our understanding of the neural underpinnings of religious beliefs (Cristofori et al., 2016; Zhong et al., 2017). Studies have indicated that reported mystical experiences are modulated by the dorsolateral prefrontal cortex (dlPFC) and middle/superior temporal cortex. Furthermore, executive functions governed by the dlPFC have been shown to play a causal role in regulating mystical experiences, with individuals who have suffered damage to the dlPFC reporting more mystical experiences, likely due to diminished executive functioning (Cristofori et al., 2016; Zhong et al., 2017). These findings support the idea that the relaxation of inhibitory networks influences certain aspects of religious experience, activating default intuitive thinking that is typically suppressed by brain regions focused on rational, analytical thinking, such as the PFC (Grafman et al., 2020). In addition, it is important to note that the dlPFC is not only essential for reducing supernatural interpretations of unusual experiences, but it does so in a context-dependent manner through cognitive control and executive functions (Andersen et al., 2019).

#### 3.1.5 Reward and evaluation

Like other belief systems, religious beliefs gain significance and influence through the prefrontal and dopaminergic circuits. Research has found that adolescents or young adults with stronger religious beliefs are more inclined to defer immediate gratification for greater future rewards (Carter et al., 2012). However, contrary to these findings, another study has indicated a positive correlation between religiosity and intertemporal discounting rates among older individuals, with higher discounting rates suggesting a quicker decline in perceived value over time (Morgan et al., 2016). Research has also indicated that the link between religious commitment and delayed gratification was influenced by future time orientation, reflecting a focus on the future and a sense of its rapid approach. Additionally, religious beliefs in the afterlife and potential future consequences may lead to a heightened awareness of the future and shape an individual’s perception of time (Carter et al., 2012). Notably, when exposed to religious statements, older adults with strong religious beliefs exhibited faster response times, and this was associated with increased fMRI functional connectivity effects among the left anterior cingulate cortex (ACC), right dlPFC, and nucleus accumbens (NAc) (Carter et al., 2012). These findings imply that religious beliefs, particularly concerning the end of life, are linked to the activation of evaluative and motivational systems that are crucial for achieving goals and obtaining rewards (Gjesme, 1979; Grafman et al., 2020).

Religious prayers can activate the reward system, a discovery that may partially explain their role in top-down control (Schjødt et al., 2008). For example, one study observed increased activity in the striatum of Danish-identifying Christians during repetitive religious prayers. This indicates that repeated prayer triggers the dopaminergic reward system, potentially motivating individuals to continue this practice (Schjødt et al., 2008). Another study investigating Mormon-identifying participants found that religious experiences influenced by ecological and religious stimuli activated various brain networks associated with reward, salience, and attention, including the NAc, ventromedial prefrontal cortex (vmPFC), ACC, and frontal attentional regions (Ferguson et al., 2018). The findings suggest that abstract religious concepts can be linked to intrinsic rewards in religious individuals, potentially reinforcing religious beliefs and behaviors (Ferguson et al., 2018).

#### 3.1.6 Conflict detection

In one study, researchers compared participants’ evaluations of religious and non-religious beliefs. The results showed that religious and non-religious propositions similarly engaged the vmPFC (Harris et al., 2009). However, religious statements uniquely activated the precuneus, ACC, insula, and ventral striatum, while non-religious statements activated the left parahippocampal, hippocampal, and temporal lobe regions (Harris et al., 2009). The activations in response to religious statements were associated with emotion, motivation, and conflict, suggesting that religious beliefs hold a distinct status in memory and behavior (Harris et al., 2009).

Theoretical frameworks, such as predictive processing, propose that monitoring errors are essential for updating and sustaining beliefs (van Elk and Aleman, 2017). A stronger belief in God is linked to decreased error-related negativity signals in the ACC when making mistakes on non-religious cognitive. This indicates that religious conviction may provide a buffer against anxiety in new situations (Inzlicht et al., 2009). How one perceives God as punitive or forgiving also impacts executive control performance. A recent study discovered that contemplating God’s perceived love led to less pronounced error-related negativity in response to mistakes and reduced vigilance for conflicts between one’s actions and religious norms (Good et al., 2015).

#### 3.1.7 Social processing

Research has shown differences in how religious- and non-religious-identifying individuals process social information in religious and non-religious contexts. During a task evaluating attractiveness, peer influence had a more substantial impact on the late positive potential than their non-religious-identifying counterparts (Thiruchselvam et al., 2017). This suggests that religious individuals may be more affected by social pressure, which can be seen in activity in the late positive potential (Thiruchselvam et al., 2017). The researchers also propose that social conformity and belonging to a particular group are essential factors in transmitting and influencing religious beliefs (Thiruchselvam et al., 2017).

Recent studies have investigated the event-related potentials (ERPs) of individuals identifying as Christian and atheist while observing images of people from the same or different religious affiliations experiencing pain. The findings revealed that there was a stronger initial frontal ERP response in individuals viewing the suffering of those with similar religious beliefs, along with a secondary heightened central/parietal empathic signal in Christian-identifying participants (Huang and Han, 2014). These results indicate that exposure to stimuli depicting individuals with shared religious beliefs may increase empathy and in-group preferences, as reflected in frontal and parietal ERPs (Huang and Han, 2014). Additionally, a study from Denmark examined the impact of assumptions about speakers’ abilities on the neural responses of Christian- and secular-identifying individuals who received intercessory prayer. The study found that assumptions about the charismatic abilities of the speakers influenced the executive function network, specifically in Christian-identifying participants, resulting in reduced front brain activity from the MPFC and dlPFC in response to charismatic speakers (Schjoedt et al., 2011). This suggests that charismatic influence may diminish PFC activity, potentially impacting trust in religious authorities and reinforcing certain expectations (Schjoedt et al., 2011).

### 3.2 Neurochemical signaling

Besides focusing on how activity in brain regions changes during engagement in religious behaviors, it is also critical to understand what is causing these changes to occur on a neurochemical level. For instance, the nervous system’s primary excitatory and inhibitory neurotransmitters, glutamate and gamma amino butyric acid (GABA), have been theorized to be essential neurotransmitters for meditative practices (Newberg and Iversen, 2003). In this hypothesis, researchers surmise that the focused attention in meditation results from the activation of the PFC by glutamate release. In turn, these glutamate neurons also project from the PFC onto the reticular nucleus of the thalamus as part of a more global attentional network (Portas et al., 1998; Zikopoulos and Barbas, 2006). When activated by glutamate, the reticular nucleus releases GABA onto the lateral posterior and geniculate nuclei of the thalamus, shutting off input to the posterior superior parietal lobe (PSPL) and visual cortex (Destexhe et al., 1998). The release of GABA in these thalamic nuclei leads to functional deafferentation, where few outside stimuli arrive at the visual cortex and PSPL, thus enhancing the sense of focus attributed to meditation, prayer, and other practices (Newberg and Iversen, 2003).

While there are contradictory findings (Mohandas, 2008) regarding the involvement of the serotonergic system with what researchers define as religious behaviors, there is some evidence that the release of serotonin and glutamate during meditation may elicit an increase in acetylcholine release to alter attentional behaviors (Borg et al., 2003; Karlsson et al., 2012). However, with psychedelic research, investigators have been able to explore how serotonin may be involved in the development of what are classified as religious experiences. In a series of studies, researchers administered psilocybin at high doses to drug-naive participants, after which they would complete a series of questionnaires designed to measure the occurrence of what are considered to be religious experiences. In these studies, nearly all participants who were administered psilocybin reported higher rates of having a religious experience (Griffiths et al., 2006; Griffiths et al., 2008; Griffiths et al., 2011; MacLean et al., 2011; Griffiths et al., 2018). Considering psilocybin’s high binding affinity for the serotonin 5-HT_2A_ receptor, researchers hypothesize that serotonin, and especially high levels of serotonergic signaling, may influence individuals’ ability to have religious experiences, whether with or without the administration of a psychoactive substance (Borg et al., 2003; Presti and Nichols, 2004; Griffiths et al., 2006).

As previously discussed regarding reward and evaluation of religious behaviors, the role of the reward system is to evaluate and motivate individuals to engage in religious behaviors (Schjødt et al., 2008; Ferguson et al., 2018). Dopamine, the primary neurotransmitter of the reward system, influences rewarding and motivating behaviors and is an important mechanism reinforcing and motivating religious practitioners to continue engaging with their faith. BOLD signals recorded from Danish-identifying Christians found increased activity in the caudate nucleus during prayer behavior, suggesting possible stimulation of the dopaminergic projections to the dorsal striatum (Schjødt et al., 2008). Other research has implicated a correlation between increased religious behaviors and motivation to a polymorphism of the dopamine receptor gene DRD4 (Comings et al., 2000; Sasaki et al., 2013).

Neurohormones have far-reaching effects in the body outside the nervous system and have also been implicated in religious behaviors (Yaden et al., 2016). One such neurohormone is oxytocin, which has been implicated in a wide range of social behaviors, such as social recognition, social learning and memory, social reward, and attachment formation, amongst others (Ferguson et al., 2001; Wang and Aragona, 2004; Young and Wang, 2004; Dölen et al., 2013; Young et al., 2014; Choe et al., 2015; Oettl et al., 2016). Heightened oxytocin levels recorded from saliva assays taken from participants who reported higher levels of spirituality suggest this neurohormone plays a role in the social cognitive aspects of religious behaviors (Bartz et al., 2011; Grigorenko, 2011; Holbrook et al., 2015). These social cognitions can include spiritual connectedness with supernatural agents, prosociality, and well-being in social affiliations (Boyer, 2007; Gutkowska and Jankowski, 2012; Holbrook et al., 2015).

Researchers have observed decreased levels of these compounds in blood serum following engagement of religious behaviors for certain neurohormones, such as epinephrine, norepinephrine, and cortisol. Epinephrine and norepinephrine regulate the body’s “fight or flight” response and have been demonstrated to have decreased levels recovered from urine and plasma samples from participants following prayer, meditation, and other calming religious practices (Newberg and Iversen, 2003). Regarding cortisol, this neurohormone is released by the adrenal gland in response to stress and has been observed to have decreased levels in serum during meditation sessions (Newberg and Iversen, 2003). The reduced levels of these neurohormones observed indicate the calming and alleviating effects that engaging with certain religious behaviors can have on individuals.

## 4 Discussion

### 4.1 Limitations

A limitation concerning NoR, which is prevalent in psychology and neuroscience in general, is the need for more diversity in subject pools. The most common religious identities investigated in NoR are either Christian or Buddhist populations (Newberg and Iversen, 2003; Yaden et al., 2016; Newberg, 2020). For starters, even amongst Christian and Buddhist populations, there can be incredible amounts of internal diversities of practice within these two broad categories, depending on regions, communities, social contexts, and cultural influences (Newberg and Iversen, 2003). However, many religious identities worldwide will have different experiences and interpretations, even if some traditions appear to share similarities. For instance, the Abrahamic religions, understood as Jewish-, Christian-, and Muslim-identifying populations, involve some affirmation of a relation to a prophetic figure, Abraham, and profess an affirmation of belief in a single deity, generally called “God,” who is considered eternal, omnipotent, omniscient, and the creator of the universe (Christiano et al., 2015). Despite this similarity, these religions differ in perceptions of such a deity. In several Jewish and Christian theological debates, God is sometimes perceived in multiple forms, whether as anthropomorphic, an animal, a voice from the sky, and much more. On the other hand, in some Islamic theological debates, God is perceived as a conscious, unseeable force behind all aspects of the universe (Leeming, 2005). Most of the world’s Christians also believe in the Trinitarian doctrine that God is a single entity existing in three divine persons: the Father, the Son, and the Holy Spirit (Prestige, 1963; Mills and Bullard, 1990; Kelly, 2014). However, amongst Jewish and Muslim theological discussions, God is understood as being strictly singular (Esposito, 1999). These are just a few examples to demonstrate that even though some religions hold similarities in their traditions, they can still have differences within those similar traditions. Considering such theological and doctrinal differences, these can affect observable differences, especially accounting for ToM conceptualizations of religion, where emphasis is placed on how participants perceive, understand, and interpret entities within their tradition. Therefore, neuroscientists must be careful when applying results collected from particular religious groups and identities to the religiously-identifying population.

As with any scientific study or field, there are limitations imposed by the technologies used. For NoR, there are limitations associated with using brain imaging and recording techniques. EEG recording suffers from meager spatial resolution, meaning recorded changes can only be localized over vast brain areas (Newberg, 2020). fMRI, the most common brain imaging technique used in NoR, has several disadvantages and limitations. The most significant concern with fMRI is that images must be obtained while the subject is in the scanner, and the scanner can make up to 100 decibels of noise (Newberg, 2020). This can create an environment that distracts the participant from performing their rituals or practices with the typical level of concentration they use for such behaviors. fMRI noise can also affect brain activity, particularly in the auditory cortex (Lazar et al., 2000; Newberg, 2020). Several investigators have attempted to address this concern and have successfully measured meditative practices in the scanner by having participants listen to fMRI noise at home. An additional limitation of fMRI studies is that they cannot evaluate individual neurotransmitter systems (Newberg, 2020). As previously discussed, neurotransmitter systems play an essential role in religious behaviors. While fMRI can locate brain regions of decreased and increased activity through blood flow, those regions comprise multiple neurotransmitter systems that cannot be differentiated, making it difficult to determine what specific neurochemical and neuronal cell populations are involved. As for PET imaging, which does permit individual neurotransmitter systems to be targeted, a significant limitation of this technique is that it generally has poor temporal resolution, meaning that it takes longer to make multiple measurements than other techniques, like EEG or fMRI (Newberg, 2020). This can create issues when recording participants during a particular moment or through different ritual states.

Of course, there is a more significant matter of concern when assessing the use of brain imaging and recording techniques within NoR that EEG, fMRI, and PET all share. This technology requires that participants be hooked up to a device, which, in the case of fMRI and PET, requires the participant’s physical presence within the apparatus. A participant must maintain limited mobility, especially in the case of fMRI and PET, both of which require the participant’s supination. This limits the types of religious behaviors participants can engage in, privileging internal, belief-centric behaviors, such as recalling religious experiences, thinking about God, praying, or meditating while stationary.

As discussed previously, ritual practice is often a co-equal component of religiosity alongside belief. Rituals and beliefs create an emotional state for individuals, allowing total investment into religious behaviors (Durkheim, 2001). Across many religions and ritualized practices, the body must move or position itself in a manner that is incompatible with brain imaging and recording machinery. One such example would include Muslims prostrating themselves in the direction of Mecca to perform *salat* (the obligatory five-times-a-day prayer in Muslim ritualizations). Another example would be prayer practices amongst specific Jewish communities praying toward Jerusalem. Given these examples of the necessity of movement in religious observation, available brain imaging and recording techniques may set an artificial and methodological limit to the development of NoR at this time. Further, given their restrictive medical settings, such techniques may also limit our ability to observe how the social aspects of religious practice, of which Durkheim wrote distinctly, affect the brain (Durkheim, 2001). Therefore, not only does this technology limit researchers in what they can explore regarding religious behaviors, but it also restricts them from fully exploring the scope of the definitions of religion they have established.

### 4.2 Future directions

#### 4.2.1 Neuroscientific and technological approaches

An interesting future direction for NoR is to incorporate research models that allow researchers to broaden how they examine religious behavior, particularly in ritualization and embodiment. In this regard, it can be helpful to look to other fields of neuroscience research, such as those examining physical activity and sports performance (Norris, 2005). One technique used to study brain activity during sports performance and physical activity that may prove helpful for researching ritualization and religious embodiment is dry electrodes during EEG recordings. Unlike the more commonly used “wet” EEG, “dry” EEG does not require applying a gel to the participant’s head before placing electrodes (Wang et al., 2019). The use of electrode gel creates time constraints on the study because it must be reapplied to the head if it dries. The need for reapplication can interrupt the subject’s activity and requires research to be done primarily in a lab setting (Wang et al., 2019). However, with dry EEG, the recording of participants can go on for extended periods and allow recording to be conducted outside of a lab setting (Wang et al., 2019). Of course, dry EEG has limitations, one of which is that it has poorer spatial resolution than wet EEG (Wang et al., 2019). This raises the question of whether this technology will provide enough additional insights to be worth using in the field. Regardless, the application of this technique should be at least explored in the future to allow researchers to record participants as they engage in religious behaviors in a religious setting.

Another technology that could prove valuable in NoR is the application of functional near-infrared spectroscopy (fNIRS). fNIRS is a non-invasive imaging technique that estimates oxygenated and deoxygenated hemoglobin levels in brain tissue by recording changes in the scattering of near-infrared light from the scalp (Obrig and Villringer, 2003). While this technology is similar to fMRI’s reliance on neurovascular coupling to make claims about brain activity, fNIRS has considerably higher temporal resolution (Kleinschmidt et al., 1996; Strangman et al., 2002; Obrig and Villringer, 2003). This higher temporal resolution could allow researchers to better record differences in brain activity and monitor changes associated with different stimuli. However, as opposed to fMRI, the most appealing aspect of fNIRS is that this technology does not require the motion of the subjects to be severely restricted (Hyde et al., 2010; Yücel et al., 2014). Like dry EEG, fNIRS is also limited, as this technology has slightly lower spatial resolution than fMRI or PET (Kleinschmidt et al., 1996; Strangman et al., 2002; Obrig and Villringer, 2003). Regarding its recording abilities, fNIRS can only measure brain activity in the outer cortical regions and not so much in the inner limbic regions (Kleinschmidt et al., 1996; Strangman et al., 2002; Obrig and Villringer, 2003). However, considering that a majority of the research in this field focuses on the PFC, this limitation may be less restricting compared to technologies like fMRI and PET (Grafman et al., 2020). Therefore, researchers could use fNIRS to record brain activity while subjects engage in more embodiment or movement-related religious behaviors, aligning with the idea established by scholars of religions that ritual and belief coincide (Durkheim, 2001; Yaden et al., 2016). Of course, if researchers want to continue exploring ritual and belief separately, they should consider incorporating works from Grace Davie into their framework, who has argued that ritual and beliefs do not always coincide, specifically with beliefs existing without the involvement of rituals (Davie, 1994).

A new direction for brain lesion and injury research involves investigating the impact of chronic traumatic encephalopathy (CTE) in postmortem studies. CTE is a neurodegenerative disease linked to repeated trauma to the head, leading to symptoms of behavioral and mood problems, and can eventually result in dementia (Stein et al., 2014; Asken et al., 2017). Investigating CTE would add to the literature present on brain lesions and injuries in religious-identifying subjects by investigating how religious behaviors may change over time in the presence of brain degeneration and offer pre- and post-trauma insights. A longitudinal study could examine religiously identifying athletes, preferably those who engaged in sports that are known for physical contact and causing head trauma. Such a study would examine religious behaviors in these athletes, measuring changes in these behaviors over time. Another method would be to perform a retrospective study, where family members of athletes are asked to report changes in religious behavior to determine if those are correlated with CTE pathology. However, a significant drawback of investigating this kind of disease is that CTE diagnoses cannot be made in living individuals and can only be determined by autopsy (Concannon et al., 2014). If researchers were to investigate the influence of CTE in changing religious behaviors, time and method of diagnosis would be factors to consider.

#### 4.2.2 Theoretical and behavioral approaches

Besides utilizing different technologies to expand the kinds of religious behaviors observed and recorded, researchers could also use different theories of religion to expand the behaviors they examine. One theorist already mentioned that has been used to formulate the current definition of religion researchers use is Émile Durkheim. While researchers currently use Durkheim’s theorization regarding belief, ritual, cohesion, and the social aspect of religion as a moral community featuring shared beliefs at its core, other aspects of Durkheim’s could be considered (Durkheim, 2001; Yaden et al., 2016). An essential element of Durkheim’s theorization of religion is his conception of the sacred and the profane and their central role in the unified systems that are religions (Durkheim, 2001). The sacred represents the interests of the group, which are set apart from mundane and individual concerns and are embodied in sacred symbols that are forbidden to be engaged with by those outside of the community (Durkheim, 2001). As for the profane, these are the mundane, individual concerns outside of what is considered sacred (Durkheim, 2001). With these ideas established, it is important to note that the sacred and profane do not necessarily mean good and evil (Pals, 1996; Durkheim, 2001). Therefore, sacred items could be good or evil if they are separate from the mundane and individual concerns that represent the group’s interests (Pals, 1996). This same principle applies to the profane, which could be good or evil (Pals, 1996). By utilizing this understanding of the sacred and profane from Durkheim, researchers in the future could record brain activity in the presence of stimuli that could be considered sacred or profane to the religious identities that a subject follows.

Another future direction to consider is the impact of authority in the presence of engagement in religious behaviors. Researchers could recruit the three ideal types of legitimate authority from the German sociologist Max Weber to draw on the conceptualization of understanding authority. Of the three ideal types of legitimate authority discussed by Weber, the categorizations of charismatic or traditional authority are most likely applicable in studying NoR (Weber, 2004). Weber describes charismatic authorities as primarily familial or religious (Weber, 2004). For traditional authorities, Weber refers to these as primarily forming out of monarchical- or feudal-like structures, where leadership is determined by customs that typically follow an individual’s lineage (Weber, 2004). Future research could present a religious authority familiar to participants as they engage in religious behaviors. Utilizing religious authorities would also expand upon the social aspects of religions as communities established by Durkheim, with these authorities serving as leaders of these communities (Durkheim, 2001).

To expand upon the concept of authorities, researchers should consider the impact of different settings as authorities while subjects engage in religious behaviors. To draw on the conceptualization of understanding different settings, researchers could recruit the ideas of hegemonic spaces and places of representation established by Henri Lefebvre. Hegemonic spaces can most typically be considered generic spaces, as they are locations we experience that are conditioned or given to us by dominant authorities (Lefebvre, 1992). These are spaces that all community members can encounter daily and are more objective in their experiences, goals, and activities (Lefebvre, 1992). By applying this understanding of hegemonic spaces, it could be argued that in the context of NoR, a lab setting, where a religiously-identifying subject is asked to engage in religious behavior, is a hegemonic space for that subject. Places of representation are specific locations that hold symbolic value and are more specific to certain communities or contexts (Lefebvre, 1992). Places of representation are also more subjective in their experiences, goals, and activities (Lefebvre, 1992). Both hegemonic spaces and places of representation are locations for spatial practice, which refers to behaviors or actions performed in a location dictated by societal and customary expectations (Lefebvre, 1992). In the future, using technologies like dry EEG and fNIRS, researchers could explore the effects of engaging in religious behaviors in places of representation, which, in this context, would be settings where subjects typically engage in the spatial practices of their religious identity. In such studies, researchers could compare the effects of engaging in religious behaviors in identified hegemonic spaces and places of representation and see if any changes occur.

To further utilize these theories in research, investigators could incorporate these concepts to establish a set and setting in a religious context for psychedelic research. In psychedelic research, set refers to the expectations, assumptions, and other pre-existing psychological factors one brings during psychedelic use, while setting refers to the environmental context in which the use is occurring (Leary et al., 1963; Hartogsohn, 2016; Hartogsohn, 2017; Carhart-Harris et al., 2018). The influence of set and setting can have a profound impact on the kinds of experiences individuals can have while under the influence of psychedelics (Carhart-Harris et al., 2018). However, while there has been much research investigating psychedelics’ ability, particularly psilocybin, to elicit what are classified as religious experiences, researchers have yet to explore the influence of set and setting in these studies (Griffiths et al., 2006; Griffiths et al., 2008; Griffiths et al., 2011; MacLean et al., 2011; Griffiths et al., 2018). For set, researchers could utilize Durkheim’s theories of ritual and belief, or the sacred and profane, to put participants into a more religious mindset before drug administration. For setting, researchers could use Lefevbre’s theories of hegemonic spaces (laboratory settings) and places of representation (religious settings) to investigate the influence of religious settings on eliciting religious psychedelic experiences.

While this paper has focused a majority of its discussion on religion under the framework of Durkheim, it is also important to consider other frameworks as well, since there is no unified definition of religion (Smith, 1982). To examine more modern interpretations of religion, there are the models proposed by Peter Berger and Zygmunt Bauman. Peter Berger proposed the idea of nomos, which refers to a culturally constructed worldview that gives meaning and coherence to the human experience (Berger, 1969). Religious beliefs and rituals help construct particular worldviews that can justify existing social orders. These external, social constructions of reality are then internalized through repeated interactions, rituals, and language, shaping how people think, feel, and act (Berger, 1969). To investigate this idea of nomos and internalization, researchers could utilize conflict detection models by presenting religious statements congruent and incongruent with their internalized nomos. As for Zygmunt Bauman, he proposed a theory of individualized religion that has risen from modern secular societies, where traditional religious institutions have lost much of their authority (Bauman, 2000). As a result, individuals no longer feel bound to inherited belief systems and can construct their own paths by selecting ideas, practices, and experiences from diverse traditions (Bauman, 2000). Bauman’s theory raises the idea for researchers to investigate those identified as religious “Nones,” individuals whose relationship with organized religions is ambiguous or unaffiliated (Zurlo and Johnson, 2016).

## 5 Conclusion

This article demonstrates that NoR uses modern neuroscience techniques to explore religious behaviors. These approaches include a host of brain imaging and recording techniques, like EEG, fMRI, and PET, as well as TBI and psychedelics. Combining these methodologies with conceptualizations of belief and ritual, researchers have implicated the PFC, the reward system, and several neurotransmitter and neurohormone systems in cognitive and motivational changes regarding engagement with religion. However, the brain imaging and recording techniques recruited in this field limit the kinds of behaviors researchers can investigate, constraining the interpretation of results and religious behaviors. Other technologies, such as dry EEG and fNIRS, can mitigate these limitations by opening new directions for exploring religious behaviors. Of course, with religion being the “object of a scholar’s study,” researchers could also expand their understanding of religion and religious behaviors through the lens of Durkheim’s sacred and profane, Weber’s charismatic authorities, and Lefebvre’s places of representation, opening further directions of investigation (Smith, 1982). By incorporating these recommended suggestions in the future, the research done in this field could start investigating new and exciting pathways to explore the neuroscience of religion.
